# Mammographic and Ultrasonographic Imaging Analysis for Neoadjuvant Chemotherapy Evaluation: Volume Reduction Indexes That Correlate With Pathological Complete Response

**DOI:** 10.7759/cureus.29960

**Published:** 2022-10-05

**Authors:** Juliana M Mello, Flavia Sarvacinski, Flavia C Schaefer, Daniel S Ercolani, Nathalia R Lobato, Yasmine C Martins, Guilherme Zwetsch, Fernando P Bittelbrunn, Charles F Ferreira, Andrea P Damin

**Affiliations:** 1 Radiology, Hospital de Clínicas de Porto Alegre (HCPA), Porto Alegre, BRA; 2 Medicine, Federal University of Rio Grande do Sul (UFRGS), Porto Alegre, BRA; 3 Physiology, Federal University of Rio Grande do Sul (UFRGS), Porto Alegre, BRA; 4 Gynecology, Federal University of Rio Grande do Sul (UFRGS), Porto Alegre, BRA

**Keywords:** breast cancer imaging, volume reduction indexes, volume reduction, breast cancer survival, cancer-specific survival, pathological complete response, ultrasound, mammography, neoadjuvant chemotherapy, breast cancer

## Abstract

Introduction: We aimed to evaluate volume reduction in digital mammography (DM) and ultrasound (US) for neoadjuvant chemotherapy (NAC) evaluation, with breast cancer-specific survival and pathological complete response (pCR) associations.

Methods: This is a retrospective observational cohort study analyzing recorded images in 122 selected subjects out of which 569 patients presented with advanced breast cancers. Spearman’s correlation and generalized estimating equations (GEE) compared volume reduction on DM and US between pCR and non-pCR after NAC with post-surgical anatomopathology. Cox regression and Kaplan-Meier curves analyzed associations between cancer-specific survival, pCR, and volume reductions.

Results: A total of 34.4% (N=42) obtained pCR and 65.6% (N=80) did not. Minimum percentage indexes needed to correlate with pCR over time were, at least, 28.9% for DM (p=0.006) and 10.36% for US (p=0.046), with high specificity (US=98%, DM=93%) but low sensitivity (US=7%, DM=18%). Positive predictive values were 82% (DM) and 86% (US) and negative predictive values were 37% (DM) and 36% (US). Cox regression and Kaplan-Meier curves demonstrated associations of breast cancer-specific survival with pCR (Cox regression coefficient {B}=0.209, CI 95%=0.048-0.914, p=0.038).

Conclusions: At least 28.9% of volume reduction on DM and 10.36% of volume reduction on US are correlated with pCR. Furthermore, pCR was associated with breast cancer-specific survival after NAC in volumetric morphological imaging analysis.

## Introduction

Patients with advanced breast cancers are considered for neoadjuvant chemotherapy (NAC) to reduce the breast tumor volume, allowing less aggressive surgical treatment and less morbidity related to more aggressive surgeries [1]. Moreover, NAC can be an in vivo parameter for tumor behavior response and it is a widespread treatment nowadays, even in smaller lesions [2]. Additionally, a better response to NAC is associated with better prognosis, longer disease-free survival, and overall survival [3].

Hence, imaging methods are crucial to predict pathological complete response (pCR) and help monitor clinical response to NAC [4,5]. The most widely accepted imaging techniques are magnetic resonance imaging (MRI) because of its higher sensitivity and specificity to predict pCR [6-8], as well as contrast-enhanced spectral mammography (CESM) [9-11]. Those techniques are more accurate than digital mammography (DM) and US mainly due to intravenous contrast injection that allows a more sensitive imaging analysis in many ways [12,13].

However, they are not generally easily accessible in extremely poor populations across the world, mainly due to higher imaging costs [14]. Even in the USA, there are some African American women and rural communities facing difficulties to engage in breast cancer treatment [15]. Saying that health insurance status may interfere in NAC response [16].

Nevertheless, it is still necessary to accurately monitor patients when treated for advanced breast cancer with NAC, despite funding restrictions. Therefore, the main objective of this study was to find minimum volume reduction percentages correlated to pCR after NAC treatment. Furthermore, the association of pCR with cancer-specific survival outcomes was tested.

Moreover, there is no standard method to monitor NAC response through imaging, especially using morphological analysis [17]. Even though, there are many advanced imaging parameters being tested especially with MRI [18], even if this is not a largely available imaging method in poorer populations, who have higher odds of mortality due to breast cancer [15].

By estimating minimum volume reduction indexes on mammography and ultrasound, this study also tried to standardize breast imaging practice to help NAC monitoring in a less expensive way, especially in areas without access to breast MRI.

## Materials and methods

A retrospective cohort study assessed institution records of 569 women who underwent NAC from January 2000 to March 2020 in a tertiary, public health, and academic hospital with a dedicated breast cancer department in Southern Brazil. Among the 569 patients with advanced breast cancers, 122 were selected through convenience for data collection and analysis, based on imaging availability on electronic hospital records, according to the inclusion and exclusion criteria listed in Table 1.

**Table 1:** Inclusion and exclusion criteria list. NAC: neoadjuvant chemotherapy

Inclusion criteria	Exclusion criteria
Patients with digital mammography (DM) and breast ultrasound (US) before the beginning of NAC	Patients without digital mammography (DM) and/or breast ultrasound (US) before the beginning of NAC
Patients with digital mammography (DM) and breast ultrasound (US) after at least two cycles of neoadjuvant chemotherapy	Patients without digital mammography (DM) and/or breast ultrasound (US) after at least two cycles of neoadjuvant chemotherapy
Patients who performed full field DM	Patients who didn’t perform full field DM
Intravenous systemic therapies administered (taxanes and/or anthracyclines)	Exclusive oral hormonal therapies, like aromatase inhibitors (anastrozole, etc.), due to clinical patients' contraindications to intravenous systemic therapies
Patients submitted to main lesion surgery excision	Patients who could not be submitted to main lesion surgery excision
Post-surgical anatomopathological analysis results are available	Post-surgical anatomopathological analysis results are not available
Advanced breast cancer (≥stage IIA)	Early breast cancer (stages 0 or I)
-	Subjects who exceeded 37% above the minimum sample size calculation (N=89)

Subjects who exceeded 37% above the sample size calculation were excluded due to funding restrictions. The sample size calculation (95% confidence interval, ɑ=0.05, β=0.80) was based on an estimated percentage of advanced breast cancers (6.15%) found in the region of Rio Grande do Sul State, Southern Brazil, considered an overall 12.9% lifetime risk of developing breast cancer, and the minimum sample size was 89 subjects [19]. The exact published numbers of breast cancer stages IIA or higher in this region were not available, but we estimated that percentage based on the 4.6% of locally advanced breast cancers estimated frequency in the United States, according to the Surveillance Epidemiology and End Results (SEER) database [20], considering that Latin America has an overall higher prevalence of more advanced breast cancer stages [21-23].

The selected 122 subjects accomplished 642 breast imaging examinations, which were analyzed before (time 1), during (time 2, after at least two cycles of chemotherapy), and after completing or interrupting NAC (time 3). The medications mostly used were Taxanes and/or Anthracyclines.

All subjects had advanced breast cancers (≥stage IIA), as proposed by the American Joint Committee on Cancer (AJCC) for more accurate death prediction related to breast cancer-specific survival [24]. All main lesion volumes were calculated based on three bigger axes at DM and US, as recommended by the Breast Imaging-Reporting and Data System (BI-RADS) [25].

All patients were offered breast surgery, including complete mastectomy or breast-conserving surgery, according to their imaging and clinical responses. All patients signed informed consent before surgery. The post-surgical anatomopathological results were considered the gold standard method to check for pCR.

The results included imaging reports from four different radiologists (with five to 20 years of experience in breast imaging) and at least two different pathologists (with more than five years of experience in breast pathology). The radiologists and the pathologists had open access to all the electronic files, including previous reports.

This study was ethically reviewed and approved by the regional ethical committee, allowing authors to collect institutional data recordings following privacy safety instructions and precautions determined by national law. Institutional ethical board (AGHUse) approval number was 2018-0397, CAAE 09778918.8.0000.5327.

The database double entry and review were performed using the SPSS, version 18.0 (SPSS Inc.: Chicago, IL). Spearman’s correlation and generalized estimating equations (GEE) compared the main lesion volume reduction on US and DM with pCR and non-pCR after NAC and post-surgical specimen analysis. The minimum percentage reduction needed to correlate with pCR was estimated in US and DM. The association between years of survival, pCR, and the estimated morphological volume indexes was analyzed using Cox regression (assessed variables were individually inserted by the enter method) and Kaplan-Meier curves.

This was a single-center study with two digital mammography equipment (Mammomat Inspiration; Siemens: Munich, Germany) and five ultrasound equipment (Philips HD15 {Philips: Amsterdam, Netherlands} and ALOKA {ALOKA, Inc.: Wallingford, Connecticut}), by the time the examinations were performed. There was less than 3% of missing at random data, which was pairwise deleted.

## Results

**Figure 1:**
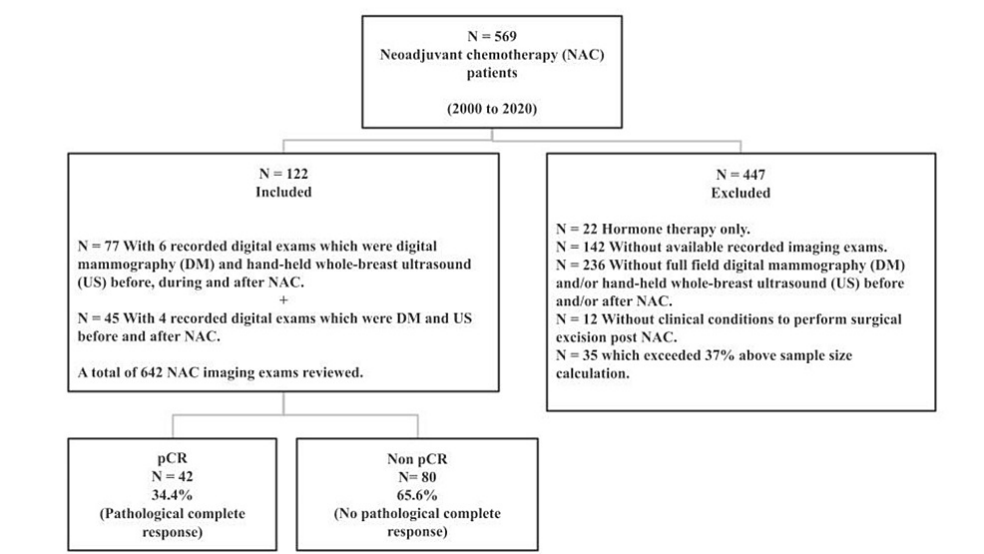
Flow diagram of study participants.

Participants flow is described in Figure 1. No patient had any known direct or prompt adverse event due to this study, as it was retrospective, observational, and conducted on available recorded medical data. Among the 122 selected patients, 34.4% (N=42) obtained pCR and 65.6% (N=80) did not accomplish pCR. The mean (±standard error of mean {SEM}) age was 48 (±0.97) years. Spearman’s correlation revealed a direct relation between pCR and the main lesion volume reduction over time in DM and US (Table 2).

**Table 2:** Spearman’s correlation showing relation between pCR and the main lesion volume reduction over time in DM and US. *Statistical significance level. ρ: Spearman’s rho coefficient

Main tumoral lesion	Pathological complete response (pCR)	
	ρ	p-Value*
Time	0.000	1.000
Volume on digital mammography (DM)	-0.291	0.006*
Volume on ultrasound (US)	-0.229	0.046*

The GEE correlating DM and US among periods of time (time 1, time 2, and time 3) and pCR after surgery demonstrated that DM main lesion volume reduction was observed when comparing times 2 and 3. On the other hand, the US main lesion volume did not show any difference over the three different periods of time. These results are detailed in Table 3.

**Table 3:** Pathological complete response correlated to DM and US volume reduction and their interaction. *Statistical difference between time. **Statistical difference between pCR. Data expressed as mean±standard error of mean. Significance was set at 5% for all analyses. GEE: generalized estimating equations; p: statistical significance index

Pathological complete response (pCR)	Digital mammography (DM)			GEE p-value*		
	Main lesion volume (mm) time 1	Main lesion volume (mm) time 2	Main lesion volume (mm) time 3	Group	Time	Interaction
No	67.4±13.9	30.1±7.6**	33.1±7.9	0.001	≤0.0001	0.245
Yes	33.8±13.9	7.2±2.1	7.0±3.0*			
Pathological complete response (pCR)	Ultrasound (US)			GEE p-value*		
	Main lesion volume (mm) time 1	Main lesion volume (mm) time 2	Main lesion volume (mm) time 3	Group	Time	Interaction
No	22.9±4.5	11.9±3.3**	11.6±4.4	0.007	≤0.0001	0.123
Yes	20.9±7.8	3.2±0.8	2.6±1.5			

The minimum percentage indexes needed to correlate with pCR over time were, at least, 28.9% for DM (p=0.006) and 10.36% for US (p=0.046). The specificity of those indexes was high (US=98%, DM=93%), but sensitivity was low (US=7%, DM=18%). Positive predictive values (PPV) were also high, from 82% (DM) to 86% (US), while negative predictive values were low (US=36%, DM=37%). Those indexes were calculated with 95% confidence intervals.

When exploring the time-related effects, survival rate was 72.9% (N=89) and death rate related to breast cancer was 13.93% (N=17) considering at least one to eight years of follow-up after NAC and surgical and radiotherapeutic interventions. There were no deaths unrelated to breast cancer during the period of the study. There were 13.11% (N=16) of subjects with loss of survival follow-up. As the missing data regarding survivorship was low (13.11%), it was considered missing at random and was pairwise deleted.

**Figure 2:**
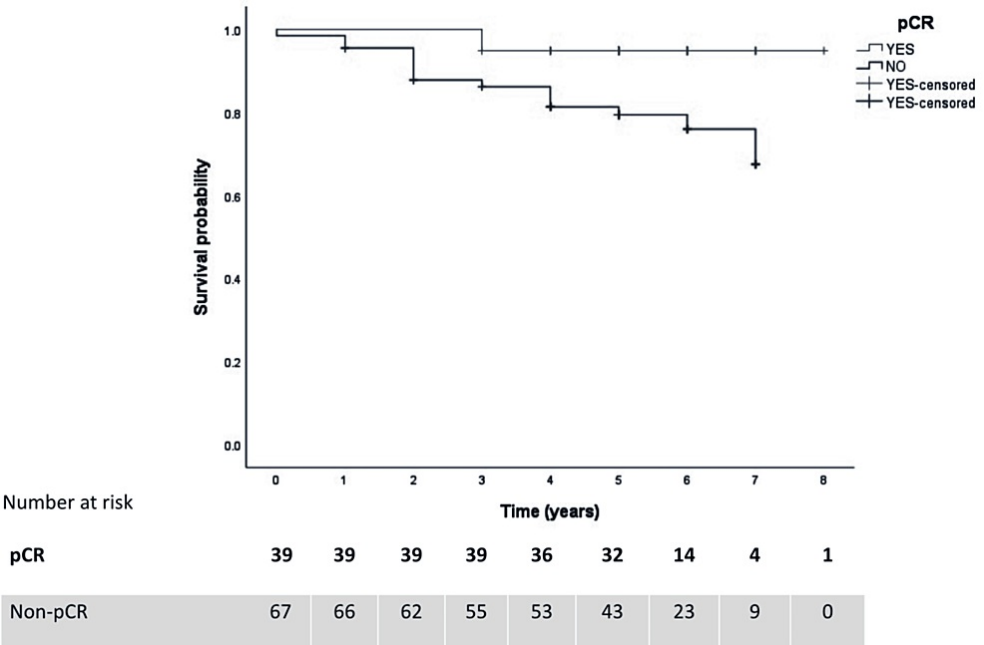
Kaplan-Meier curves explaining the association of pCR with breast cancer-specific survival outcome. pCR: pathological complete response

For the association of DM volume reduction (≥28.9%) with years of survival, Cox regression was not statistically significant (Cox regression coefficient {B}=0.925, CI 95%=0.261-3.271, p=0.903). The association of US volume reduction (≥10.36%) with years of survival was also not significant (Cox regression coefficient {B}=0.535, CI 95%=0.121-2.365, p=0.409). Kaplan-Meier curves demonstrated the association of pCR with more years of breast cancer-specific survival (B=0.209, CI 95%=0.048-0.914, p=0.038), as demonstrated in Figure 2.

## Discussion

Although Spearman's correlation degree found was weak, the results demonstrate the importance of DM and US volume reduction measurements at institutions where breast MRI is not widely available to all patients. It may help radiologists work with an alternative statistically significant way of following breast cancer NAC through imaging, although not as accurate as breast MRI, CESM, or FDG-PET, which are more advanced and contrast-enhanced techniques [26]. However, MRI major sensitivity on disease extension, multifocal disease, and nodal involvement detection when compared to non-MRI imaging techniques has led to more aggressive surgical management and no difference on five years disease-free survival outcomes [27].

At present, there are still missing minimum volume indexes correlated to pCR after NAC on DM and US evaluation [5]. The evaluations made on volume variations are mostly guided by subjective considerations, not objective quantitative standardized minimum indexes. Even the Breast Imaging-Reporting and Data System (BI-RADS) does not have a patronization on how to report NAC imaging evaluation in DM and US, although mammography remains the gold standard screening method for breast cancer [25].

By now, only the Response Evaluation Criteria In Solid Tumors (RECIST) has suggested 30% of major measurement reduction after NAC as partial response. Complete response is considered if no main lesion is founded after treatment and less than 30% response is considered a non-response. On the other hand, RECIST was not created for mammography and ultrasound breast cancer lesions’ evaluations, but mainly for metastatic measurements after systemic solid tumors treatments [5]. Nevertheless, there is scientific need for standardization of volume imaging evaluation in poorer regions across the globe, where breast MRI has more difficulties to be widely implemented.

The specificity of those indexes was high (US=98%, DM=93%), even when compared to the MRI results explored in a recent meta-analysis, demonstrating an average specificity of 81.3% [7]. Nevertheless, the sensitivity of the indexes tested in this study was low (US=7%, DM=18%), especially when compared to MRI results (sensitivity: 84.1%) in the same meta-analysis [7].

There are some limitations to this study, besides evaluating retrospective and observational data, which can lead to more difficulties when aggregating data records. One of the limitations is that mammography volume reduction percentage indexes can only be measured when main cancer lesions are big enough to be detected on mammography. Furthermore, usually bigger lesions are also related to worse prognosis. Thus, this study's conclusions may not be applicable in stage 0 or stage I lesions that may not be visible on mammography, depending on overall breast parenchymal density [28].

Moreover, another limitation to consider is that ultrasound evaluation is an essential operator dependent on results and not all radiology centers have trained radiologists to perform breast cancer ultrasounds [29]. However, ultrasound remains the most accessible and low-cost imaging evaluation method in low-income regions for dense breasts.

The implications of this study for clinical practice are more useful in low-income regions, indicating more specific morphological indexes in NAC follow-up evaluation. In centers where scientific research and finances are more promising, adding artificial intelligence (AI) could more accurately calculate the reduction of the main lesion's volume on standard full-field digital mammography and US and reinforce the results expressed above [29,30].

This study highlights the estimated main lesion volume reduction on DM and US as potential imaging indexes for pathological complete response after neoadjuvant chemotherapy, whose imaging analysis is not yet standardized on DM and US evaluation. The study demonstrates this procedure can improve the morphological imaging evaluation effectiveness of advanced breast cancer treatment in deprived areas.

## Conclusions

Supported by this study, it is concluded that a reduction of at least 28.9% of the main lesion volume on DM or at least 10.36% reduction of the main lesion volume on US are correlated to pCR. Those minimum volume reduction indexes do not assure pCR but they can support greater probability of pCR in clinical practice.

More prospective and multicentric studies are necessary to confirm and generalize the results found as possible morphological imaging indexes to predict pCR after NAC in places where breast MRI and CESM are not widely available. Even when interpreting breast MRI NAC results, recognizing morphological imaging indexes as a complementary analysis may allow more precise imaging results evaluation.
